# Sterilization of Dental Instruments

**Published:** 1891-09

**Authors:** 


ARTICLE III.

### STERILIZATION OF DENTAL INSTRUMENTS.

The last decade has taught the world more concerning true surgical pathology than all the twenty-three hundred years beside that have elapsed since the days of Hippocrates, the Father of Medicine. The eighth decade witnessed wonderful advances, while the ninth has given us a fair discernment of that which the eighth but ushered in. What shall the teeth, upon which we have now fairly entered, bring to us?

In all probability, one thing will be a more perfect comprehension of how best to secure a state of asepticism, or of freedom from pathogenic, disease-producing bacteria. Miller has shown us in his latest writings that the source of infection is not the air. He makes an elaborate computation to demonstrate that only once in ten thousand times could a cavity, the size of the usual opening into a pulp chamber, be infected from germs floating in the atmosphere. It is from the fluids of the mouth, and from infected instruments, that septic inoculation takes place. Every unclean bur and every foul excavator may carry with it into the mouth a disease-producing organism. The patient who visits the dentist in search of health may actually be infected with loathsome disease instead.

Nor is the danger very remote. Any dentist who will keep his eyes open, and who is sufficiently intelligent to recognize the symptoms of septic infection, has seen cases in which pyorrhœa, and other communicable diseases, have actually been the result of a visit to the dentist. Instances are not at all unknown in which innocent people have acquired the primal syphilitic lesion from a dentist's instruments.

Read the tale of some of Miller's experiments, you who would treat this as a light matter, and learn how he has inoculated mice and other animals by simply making a slight puncture with the point of an instrument infected from a mouth, the possessor of which probably supposed it to be in a decently cleanly state, and how the animals exhibited all the peculiar symptoms of the septic condition, constantly aggravated until death closed the scene. Then recollect that an instrument in your hands, and in a similar plight, might have infected some fair and guileless maiden who had intrusted herself to your care, had it been used in her mouth and the tissues been never so slightly wounded by it. Will not the conscientious practitioner tremble at the risks he may have run, and groan in spirit at the thought of the injury he may have inflicted? But what is to be said of the dentist who is yet recklessly rushing on in his filthy ways, regardless of the great harvest of disease which must be garnered from the seeds he is so widely sowing.

But how is the conscientious dentist, who is honestly striving to give a fair equivalent for the fees he is receiving—how is he to avoid becoming a curse instead of a blessing?

In the first place, he can observe decent cleanliness, at least. It is well known that filth and dirt are the breeding places of disease germs. He can clean his instruments after every operation, never allowing them to go from one mouth to another without careful washing. That will remove many of the risks, and if a mouth be specially bad he can be specially particular in his purification. The very thought of carrying matter from the dirty teeth of some diseased roue to that of a pure young lady is——augh!

But mere washing is not enough. No general surgeon of the present day would think of using instruments that had not been submitted to aseptic precautions. At the operating table they are kept in an antiseptic solutions. The surgeon who would neglect this would not be recognized by his brethren.

Perhaps these extreme prophylactic measures are impracticable for the dentist. He may not be called upon to do more than thoroughly to clean his instruments after each patient, unless there are indications of septic conditions. But if he does not sterilize them after working in septic canals, after opening abscesses, after operating in a mouth infected with pyorrhœa, and especially in one that bears any indication of erosions that may by any possibility be of a specific character, he is unfit to have anything to do with the mouth of another human being.

How shall this sterilization be accomplished? Miller has shown in his latest writings that sterilizing fluids cannot be depended upon. A solution of carbolic acid, trichlorphenol, and even of mercuric chloride, proved absolutely unreliable, unless the instruments were allowed to remain in it for hours, and that of course is impracticable. Hundreds of experiments showed that a bur, for instance, though left to soak for a long time in a solution of mercuric chloride, might not have the germs destroyed, because they would be deeply buried beneath the debris that would prevent the solution from reaching them.

The only thing that proved efficacious was first, the mechanical cleaning with a brush of all instruments like burs, and then their submersion for from three to five minutes in boiling water. This was reasonably sure death to all pathogenic or disease-producing organisms.

But boiling water is apt to rust instruments. It does not draw the temper from steel, but it tarnishes it, at least. This may be partially remedied, and the sterilization be made more complete, by adding a little carbonate of soda to the water.

We have for some time been using an apparatus originally devised for the sterilization of milk and other foods. It consists of a chamber, which is enclosed within a protecting jacket. Within this is a perforated tray, upon which instruments can be placed. A double water receptacle in the bottom opens by a comparatively small aperture into the sterilizing chamber, and a gas burner or lamp beneath it causes the water to boil violently at the opening and to give off steam. This being confined within the double chamber can be made even superheated, and it is to this steam that the infected instruments are submitted for a few moments. When removed and wiped, they are sufficiently hot to become entirely dry in a moment.

The apparatus is made of tin and copper, and is a modification of what is known as "The Arnold Steam Sterilizer." Its cost is only between two and three dollars. —*Dental Advertiser*.

